# Physico-chemical properties of instant ogbono (Irvingia gabonensis) mix powder

**DOI:** 10.1002/fsn3.220

**Published:** 2015-03-09

**Authors:** Oluwaseun P Bamidele, Omotayo S Ojedokun, Beatrice M Fasogbon

**Affiliations:** Department of Food Science and Technology, Obafemi Awolowo UniversityIle-Ife, Nigeria

**Keywords:** Acceptability, dika kernel powder, ingredients, nutrient, *Ogbono* soup, processing

## Abstract

The main objective of the research is to develop a recipe of instant dry soup mix for easy preparation of *ogbono* soup. Instant *ogbono* mix powder was processed using common locally ingredients. Dika kernel powder, dried *ugwu* leaf, crayfish, stock fish, and a mixture of locust bean, onion, seasoning and Cameroon powder were formulated at different ratios to find the best acceptable ogbono mix powder. The samples were subjected to proximate, functional, vitamin, mineral, and sensory analyses. The formulated sample D with the highest ratio of crayfish and stock fish had the highest value of protein and carbohydrate (24.13 and 35.61%, respectively). The control sample (100% dika kernel powder) was low in moisture content (6.20%) but high in crude fat, other samples followed in this order (control > A > B > C > D) for crude fat. Ash, crude fiber, and carbohydrate showed a significant difference (*P *<* *0.05) in all the samples. The functional properties of the sample showed a significant difference (*P *<* *0.05) in all the samples with the control having the highest value for the water absorption, swelling capacity, and bulk density which may be due to the high crude fiber and low moisture content recorded for the control sample in the proximate analysis. The mineral content of all the samples were higher than the control with phosphorous having the highest value and iron the least value. Vitamin C was the main dominating vitamin in the sample followed by vitamin B_2_, vitamin A, and vitamin B_3._ The sensory evaluation revealed that 100% dika kernel powder gave a good attribute of the soup but with less nutritional composition, while some formulated samples showed a similar attribute with higher nutritional value. Sample A with the highest overall acceptability had the best attribute of *ogbono* soup. Instant *ogbono* mix powder has higher nutritional value and easy to cook.

## Introduction

Seeds are prominent features in the peasant dietary, especially in the developing countries and oilseeds are becoming valuable sources of nutrient for man, especially in countries where the diet is plant based. Ignorance of their food value has resulted in their wastage in terms of economic returns or postharvest loses. Much work has been done on some Nigerian seeds and legumes including *Irvingia gabonensis* seeds. The dika tree (*Irvingiacea* spp) is very valuable for its edible yellow mango-like fruit and termite-resistant wood (Harris 1996 and Ayuk et al. 1999). There are two common species, *Irvingia gabonenesis* that has a sweet edible pulp and *Irvingia wombolu*, which has a bitter pulp. The kernel from both species has been found useful because of their valuable food properties (Omogbai 1990 and Ejiofor 1994). It constitutes an important part of the natural diet in West Africa for controlling dietary lipids and weight gain (Leakey et al. 2005; Ngodi et al. 2005 and Ogunsina et al. 2008). The nutritional composition of dika seed indicated that it contains 8.65% protein, 14.1% carbohydrate, 2.1% moisture, 1.4% crude fiber, 16.8% ash, and 38.9% dietary fiber (Eka 1980 and Okolo 1987). Solvent extraction of *Irvingia gabonensis* seed yielded between 68 and 75% fat (Onwuka et al. 1984), this showed that the seed is an oil seed.

Powdered dika kernel is commonly cooked with vegetables into *ogbono* soup, which is the most important soup in Nigeria and common in the South East of Nigeria and some West African countries like Gabon and Ghana (Eka 1980). One of the major setbacks of the soup is the stress involved in getting all the ingredients like vegetable, crayfish, stockfish, etc.; the special skills required in cooking the soup; and exploiting the full nutrients of the soup which includes long cooking time. This study is therefore aimed at reducing the stress involved in cooking the soup by providing instant *ogbono* soup mix powder that contains nearly all the ingredients except meat and oil.

## Materials and Methodology

Dika kernel (*ogbono*), stock fish, cray fish, Cameroon pepper, *ugwu* leaves, salt, seasoning, and locust bean were procured from Oja tuntun market in Ile-Ife, Osun State, Nigeria. The dika kernel, cray fish, stock fish, locust bean, and Cameroon pepper were processed into powdery form. The ugwu leaves were shredded, washed, blanched, drained, and dried. There were four different formulation of samples; (*ogbono* powder: Crayfish: Stock fish: *Ugwu*: (mix of locust bean, onion, seasoning, and Cameroon powder)) Control – 100% dika kernel powder, sample A- 65: 5: 15: 5: 10, sample B- 55: 10: 15: 5: 15, sample C-50: 10: 20: 10: 10, sample D- 30: 20: 20: 10: 20. Analyses were carried out on all the powdered samples in triplicates.

### Proximate composition

The moisture content, crude protein, crude fat, ash, and crude fiber of all the samples were determined according to the method of AOAC (2000), carbohydrate contents were determined through difference.

### Functional properties determination

#### Water absorption determination

The water absorption was determined by a modified method of Lin and Zayas (1987). Each sample (2.0 g) was transferred into lagged 50 mL centrifuge tube and weighed (*W*_1_). Exactly 30 mL of hot distilled water (70°C) was added to each sample gently to wash down samples at the sides of the centrifuge tubes using a glass stirring rod. The sample and the water was mixed for 30 min. The suspension was allowed to rest for 10 min, the flour adhering to the side of the centrifuge was scrubbed down with glass rod to prevent it from drying. Additional 10 mL of hot distilled water was used to wash the sample adhering to the stirring rod into the sample. The suspension was centrifuged at 1165× ***g*** for 25 min at 50°C The tube was cooled in a desiccator and weighed (*W*_2_).



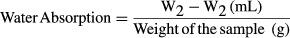


#### Bulk density determination

Bulk density was determined using the gravimetric method as described by Okaka and Potter (1976). The sample (10 g) was weighed into a 25 mL graduated cylinder. The cylinder was gently tapped ten times against the palm of the hand. The bulk density was expressed as the sample per volume occupied by the sample.

#### Swelling capacity determination

This was done using the modified method of Lin and Zayas (1987). Each sample (2 g) was dispersed in 40 mL distilled water. The resultant slurry was heated at a temperature of 70°C for 30 min in a water bath, cooled to room temperature, and centrifuge at 598× ***g*** for 30 min. The supernatant liquid was decanted and the centrifuge tube was placed in a hot air oven and dry for 25 min at 50°C. The residue was weighed (*W*_2_). The centrifuge tube containing the sample alone was weighed prior to the addition of distilled water (*W*_1_).



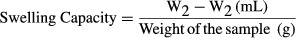


### Mineral analysis

Minerals (potassium, sodium, calcium, iron, and phosphorous) of the instant *ogbono* soup mix were determined employing the AOAC (2000) method on digestion of the sample with a mixture of concentrated nitric acid, sulphuric acid, and perchloric acid (10:0:5:2, v/v) using an atomic absorption spectrophotometer (GBC 904AA; Germany). The total phosphorus was determined as orthophosphate by the ascorbic acid method after acid digestion and neutralization using phenolphthalein indicator and combined reagent. The absorbance was read at 880 nm (Spectronic 21 D, Miltonroy, NY) and KH_2_PO_4_ (Merck, India Limited, Mumbai, India) served as the standard.

### Vitamins analysis

Ascorbic acid was extracted and estimated according to the method described by Sadasivam and Manickam (1996). About 3 g of each sample was mixed with 25 mL of 4% oxalic acid and filtered. Bromine water was added drop by drop to 10 mL of the filtrate until it turned an orange-yellow to remove the enolic hydrogen atoms. The excess of bromine was expelled by blowing in air. This filtrate was made up to 25 mL with 4% oxalic acid and used for ascorbic acid estimation. About 2 mL of the extract was made up to 3 mL with distilled H_2_O in a test tube. Another 1 mL of 2% 2, 4-dinitrophenyl hydrazine reagent and a few drops of thiourea were added. The contents of the test tube were mixed thoroughly. After 3 h incubation at 37°C, 7 mL of 80% H_2_SO_4_ was added to dissolve the osazone crystals and the absorbance was measured at 540 nm against a reagent blank. The ascorbic acid content present in the sample was calculated by referring to a standard graph and expressed as milligrams per 100 grams of powdered samples.

For the extraction of riboflavin and niacin, method of AOAC (2000) was used. Content present was calculated by comparison with a standard curve developed using standards for vitamins, such as thiamine hydrochloride, riboflavin, and nicotinic acid, purchased from sigma St, Louis, USA.

Vitamin A in form of *β*-Carotene was determined using the method of AOAC (2000), which involved extraction of the samples with acetone and hexane. Concentrations were determined by comparison with a standard curve developed using *β*-Carotene from Sigma St, Louis.

### Sensory evaluation of the soup mix

**Figure 1:**
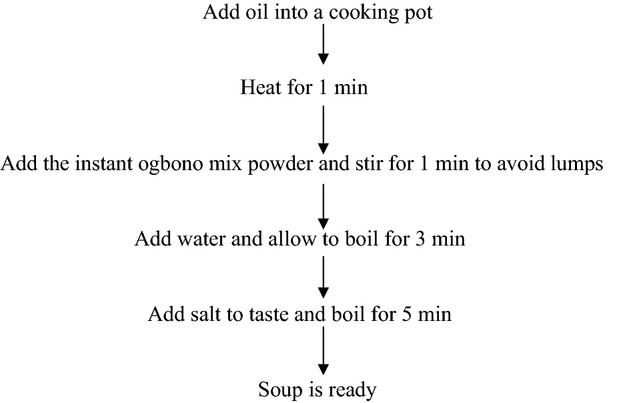
Flow chart for the preparation of ogbono soup.

The instant *ogbono* mix powder was processed into soup as shown in Figure1. Samples were coded alphabetically and presented to twenty untrained panels but lovers of “*ogbono*” soup to test for the following attributes: color, flavor, taste, texture (slick/slippery) and overall acceptability. The panelists were provided with a mouth rinse between each tasting. The attributes were scored using a 9-point hedonics scale (nine equals like extremely and one equals dislike extremely).

### Statistical analysis

Each analysis was carried out in triplicate. Data obtained from the phyisco-chemical and sensory analyses were subjected to analysis of variance (ANOVA) and the means were separated by the lowest standard deviation test (SPSS 16.0 2008). Significant level was accepted at 5%.

## Result and Discussion

### Proximate composition

The proximate composition of the formulated *ogbono* soup mix is shown in Table1. The moisture content of all the formulated samples was higher than that of the control (100% dika powder). The value ranged from 9.60 to 14.36%, while the control sample was 6.20%. The increase in moisture content of the formulated samples may be due to addition of other ingredients. The protein content has the same trend ranged between 18.42 to 24.13% for formulated samples and 10.40% for control sample. Sample D had the highest protein which may be due to the present of larger percentage of crayfish and stock fish which are major protein in nutritional composition. The rest samples followed the same trend because of the crayfish, stock fish and other ingredients added. Limited work has been done in regard of the increase in protein content, Ekpe et al. (2007) reported increased protein content as a result of the processing method of fermentation of the seeds, but the facts still remain that the ratio of other ingredients added to the soup mix still contributed to the high nutritional composition of the samples.

**Table 1:** Proximate composition of different formulation of Instant “Ogbono” powder mixed soup (%)

Sample	Moisture	Protein	Fat	Ash	Crude fiber	Carbohydrate
Control	6.20^e^ ± 0.41	10.40^e^ ± 0.20	56.41^a^ ± 0.41	8.16^b^ ± 0.10	1.52^a^ ± 0.20	17.31^e^ ± 0.40
A	9.60^d^ ± 0.30	18.42^d^ ± 0.17	34.62^b^ ± 0.21	9.20^a^ ± 0.12	0.98^b^ ± 0.40	27.18^d^ ± 0.15
B	14.36^a^ ± 0.25	20.51^c^ ± 0.30	28.40^c^ ± 0.14	7.41^c^ ± 0.30	0.51^d^ ± 0.11	29.17^c^ ± 0.14
C	11.40^c^ ± 0.20	21.51^b^ ± 0.10	25.40^d^ ± 0.16	8.23^b^ ± 0.50	0.26^e^ ± 0.12	33.20^b^ ± 0.10
D	12.40^b^ ± 0.20	24.13^a^ ± 0.12	20.13^e^ ± 0.20	6.98^d^ ± 0.17	0.75^c^ ± 0.15	35.61^a^ ± 0.23

A: Dika nut powder (65%), Crayfish (5%), Stock fish (15), Ugwu (5%), mixture of locust bean, onion mix, seasoning and Cameroon powder (10%).

B: Dika nut powder (55%), Crayfish (10%), Stock fish (15), Ugwu (5%), mixture of locust bean, onion mix, seasoning and Cameroon powder (15%).

C: Dika nut powder (50%), Crayfish (10%), Stock fish (20), Ugwu (10%), mixture of locust bean, onion mix, seasoning and Cameroon powder (10%).

D: Dika nut powder (30%), Crayfish (20%), Stock fish (20), Ugwu (10%), mixture of locust bean, onion mix, seasoning and Cameroon powder (20%).

Control: 100% Dika nut (ogbono) powder.

Values are means and standard deviation of three determinations (*n* = 3).

Means followed by the same letter within the same rows are not significantly (*P* > 0.05) different according to the LSD test.

The crude fat content of the control sample was higher when compared to the formulated samples, this is because dika seed has been classified as an oil seed (Idowu et al. 2013). 56.41% crude fat was recorded for the control sample followed by sample A, B, C, and D (34.62, 28.40, 25.40 and 20.13%) respectively. Crude fat increased with increase in dika kernel powder. These findings was supported by the work of Ekpe et al. (2007) and Ekundayo et al. (2013) on proximate composition and amino acid profile of dika nut seed and effects of microbial fermentation of bush mango/dika seed cotyledon, where both author established high content of crude fat in the seed.

Sample A had the highest ash content (9.20%), no significant difference (*P* < 0.05) in the ash content of sample C and the control sample (8.23 and 8.16%, respectively). Lower values were recorded for sample B and D (7.41 and 6.98%), respectively. The values obtained are quite higher than those reported by Idowu et al. (2013) and this may be as a result of the kernels used in this study which are nondefatted. Crude fiber contents of all the samples were lower than the control. Report has shown that low crude fiber is nutritionally valued as it traps less protein and carbohydrate (Balogun and Fetuga 1986). The carbohydrate value of all the samples were higher than the control and this may be due to the nutritional composition of dika nut seed which was reported to be oil seed majorly (Ekpe et al. 2007). Also the formulated samples recorded higher value of carbohydrate based on the carbohydrate content of the ingredients added.

### Functional properties

The functional properties of the samples are shown in Table2. The control sample had the highest water absorption capacity followed by sample A, B, C and D (9.85, 9.22, 8.73, 8.15 and 4.71%), respectively. This result followed the trend of crude fat content of the samples. The increase in water absorption capacity is due to the increase in dika kernel powder added to each sample. This result is in consonant with the finding of Ndjouenkeu et al. (1997) who reported using dika nut flour as a thickener in soup because of its higher water absorption power. The swelling capacity of the samples also followed the same pattern with water absorption capacity with control having the highest and sample D having the lowest (Control > B > A > C > D). The bulk density value varied from one sample to another. Although the control had the highest value and sample C with the lowest. There was no significant difference (*P* < 0.05) between samples A, C and D, but the simulated samples are significantly (*P* < 0.05) different from the control sample. This may be due to the proportion of some ingredients added.

**Table 2:** Functional properties of different formulations of Instant “Ogbono” powder mixed soup

Sample	Water absorption	Swelling capacity	Bulk density
Control	9.85^a^ ± 0.20	12.04^a^ ± 0.50	4.27^a^ ± 0.10
A	9.22^b^ ± 0.61	10.92^c^ ± 0.71	3.75^c^ ± 0.05
B	8.73^c^ ± 0.59	11.78^b^ ± 0.18	4.05^b^ ± 0.10
C	8.15^d^ ± 1.31	8.69^d^ ± 0.39	3.53^e^ ± 0.20
D	4.71^e^ ± 0.15	5.66^e^ ± 0.39	3.60^d^ ± 0.10

Values are means and standard deviation of three determinations (*n* = 3).

Means followed by the same letter within the same rows are not significantly (*P* > 0.05) different according to LSD test.

### Mineral content

Potassium, Sodium, Calcium, Iron and Phosphorous were found present in the control and formulated samples of the instant *ogbono* mix powder as shown in Table3. All the formulated soup mix samples are richer in minerals content compared to the control. Sample C and sample D had the highest potassium content (371.30 and 374.54 mg/100 g, respectively) followed by sample A and sample B (293.77 and 295.39 mg/100 g, respectively) compared to the control (59.70 mg/100 g). For Sodium, values of all the formulated samples ranged between 226.70 and 254.80 mg/100 g while the control is 2.00 mg/100 g. Calcium was 76.50 mg/100 g for control and ranged from 78.39 to 94.69 mg/100 g for the formulated soup mix samples. The calcium content for the control sample is in close range to the other samples. The iron contents of all the samples including the control were relatively small compared to other minerals. Iron content of the control was 2.40 mg/100 g and the other samples ranged from 4.77 to 8.09 mg/100 g. Phosphorous had the highest value among all the mineral content analysed, the control had the least value of 0.26 mg/100 g while the formulated samples value ranged between 672.40 and 1272.10 mg/100 g. This result was in line with the findings of Kayode et al. (2010) who reported high value of phosphorous in some traditional soups analysed. High result in the mineral content of all the formulated samples showed the effect of other ingredient added to them. The mineral contents are known to be good for tissue functioning and they are also necessary as a dietary requirement for human nutrition and growth (Soetan et al. 2010).

**Table 3:** Mineral content of instant “Ogbono” powder mixed soup (mg/100 g)

Samples	K	Na	Ca	Fe	*P*
Control	59.70^e^ ± 0.50	2.00^e^ ± 0.30	76.50^e^ ± 0.12	2.40^e^ ± 0.10	0.26^e^ ± 0.22
A	293.77^d^ ± 0.51	252.38^b^ ± 0.20	94.69^a^ ± 0.10	8.09^a^ ± 0.20	1272.10^a^ ± 0.30
B	295.39^c^ ± 0.30	238.33^c^ ± 0.50	90.19^b^ ± 0.22	7.03^b^ ± 0.10	1094.70^b^ ± 0.14
C	371.30^b^ ± 0.30	254.80^a^ ± 0.15	87.39^c^ ± 0.30	6.88^c^ ± 0.44	1027.20^c^ ± 0.10
D	374.54^a^ ± 0.10	226.70^d^ ± 0.11	78.39^d^ ± 0.40	4.77^d^ ± 0.50	672.40^d^ ± 0.12

Values are means and standard deviation of three determinations (*n* = 3).

Means followed by the same letter within the same rows are not significantly (*P* > 0.05) different according to LSD test.

### Vitamins

Table4, summarizes the vitamin content of the samples. Vitamin A content ranges from 0.01 mg/100 g for control to 3.15 mg/100 g for sample D. Vitamin B_2_ contents of all the formulated samples were higher (12.25–30.25 mg/100 g) than that of the control (0.01 mg/100 g) Vitamin B_3_ ranged from 0.05 mg/100 g for control to 0.20 mg/100 g for sample D. The control sample recorded the lowest value of all the vitamins except that of vitamin C with the highest value of 62.54 mg/100 g. Sample D recorded the least vitamin C (22.14 mg/100 g). The results showed that vitamin C is high in dika kernel, but lower values were recorded for formulated samples because the ratio of dika kernel powder reduced from Sample A down to Sample D. The result also showed that instant *ogbono* soup powder is a good source of Vitamin B_2_ and Vitamin C. The increase in Vitamin B_2_ content of all the samples may be due to addition of fluted pumpkin (*Ugwu*) leaf which is believed to be rich in vitamins (Adumanya et al. 2012). Vitamin C is an essential element in collagen formation and it strengthens antioxidant for resistance to infection, and also improves the absorption of Iron.

**Table 4:** Vitamin content of instant “Ogbono” powder mixed soup

Samples	Vitamin A	Vitamin B_2_	Vitamin B_3_	Vitamin C
Control	0.01	0.01	0.05	62.54
A	1.98	30.25	0.18	40.72
B	2.00	25.42	0.15	34.12
C	2.20	22.12	0.15	30.56
D	3.15	12.25	0.20	22.14

Values are means and standard deviation of three determinations (*n* = 3).

Means followed by the same letter within the same rows are not significantly (*P* > 0.05) different according to LSD test.

### Sensory evaluation

Result of the sensory evaluation is shown in Table5. All samples were prepared into *ogbono* soup and served to twenty untrained panelists but lovers of the soup to judge the samples based on specific parameters like color, taste, texture, flavor, and overall acceptability. Samples A, B, C and Control have similar values for color while sample D showed the least value because of the dark color of the pepper used. The same trend was followed for flavor where sample C and D gave the least value and the other samples including control had highest values. Sample A scored the highest in taste followed by sample C. The other samples showed similar values with the control sample and this may be due to the amount of other ingredients added with attention placed on crayfish and stock fish. Sample B had the least result of texture while sample A and control showed the highest value due to the quantity of dika kernel powder in them. Sample A and Control were rated the highest on overall acceptability based on the fact that they showed the real attribute of *ogbono* soup that people like which is attributed to the quantity of dika kernel powder added to the sample.

**Table 5:** Sensory evaluation result of Instant “Ogbono” powder mixed soup

Samples	Color	Flavor	Taste	Texture	Overall acceptability
Control	5.26	5.45	4.80	5.65	6.07
A	5.20	5.40	5.70	5.60	6.10
B	5.06	5.00	4.75	3.50	5.30
C	5.00	4.60	5.30	4.60	4.60
D	3.60	4.50	4.80	4.30	5.69

Values are means and standard deviation of three determinations (*n* = 3).

Means followed by the same letter within the same rows are not significantly (*P* > 0.05) different according to LSD test.

## Conflict of Interest

None declared.
